# Magnetic Nanoparticles: From Design and Synthesis to Real World Applications

**DOI:** 10.3390/nano7090243

**Published:** 2017-08-29

**Authors:** Jiri Kudr, Yazan Haddad, Lukas Richtera, Zbynek Heger, Mirko Cernak, Vojtech Adam, Ondrej Zitka

**Affiliations:** 1 Department of Chemistry and Biochemistry, Mendel University in Brno, Zemedelska 1, CZ-61300 Brno, Czech Republic; george.kudr@centrum.cz (J.K.); yazanhaddad@hotmail.com (Y.H.); oliver@centrum.cz (L.R.); zbynek.heger@mendelu.cz (Z.H.); vojtech.adam@mendelu.cz (V.A.); 2 Central European Institute of Technology, Brno University of Technology, Technicka 3058/10, CZ-61600 Brno, Czech Republic; 3 CEPLANT R&D Centre for Low-Cost Plasma and Nanotechnology Surface Modifications, Masaryk University, Kotlarska 2, CZ-61137 Brno, Czech Republic; cernak@gimmel.ip.fmph.uniba.sk

**Keywords:** magnetic resonance imaging, nanocarrier, nanoscale, preconcentration, separation, silica, theranostics, therapeutic agents

## Abstract

The increasing number of scientific publications focusing on magnetic materials indicates growing interest in the broader scientific community. Substantial progress was made in the synthesis of magnetic materials of desired size, morphology, chemical composition, and surface chemistry. Physical and chemical stability of magnetic materials is acquired by the coating. Moreover, surface layers of polymers, silica, biomolecules, etc. can be designed to obtain affinity to target molecules. The combination of the ability to respond to the external magnetic field and the rich possibilities of coatings makes magnetic materials universal tool for magnetic separations of small molecules, biomolecules and cells. In the biomedical field, magnetic particles and magnetic composites are utilized as the drug carriers, as contrast agents for magnetic resonance imaging (MRI), and in magnetic hyperthermia. However, the multifunctional magnetic particles enabling the diagnosis and therapy at the same time are emerging. The presented review article summarizes the findings regarding the design and synthesis of magnetic materials focused on biomedical applications. We highlight the utilization of magnetic materials in separation/preconcentration of various molecules and cells, and their use in diagnosis and therapy.

## 1. Introduction

**Figure 1:**
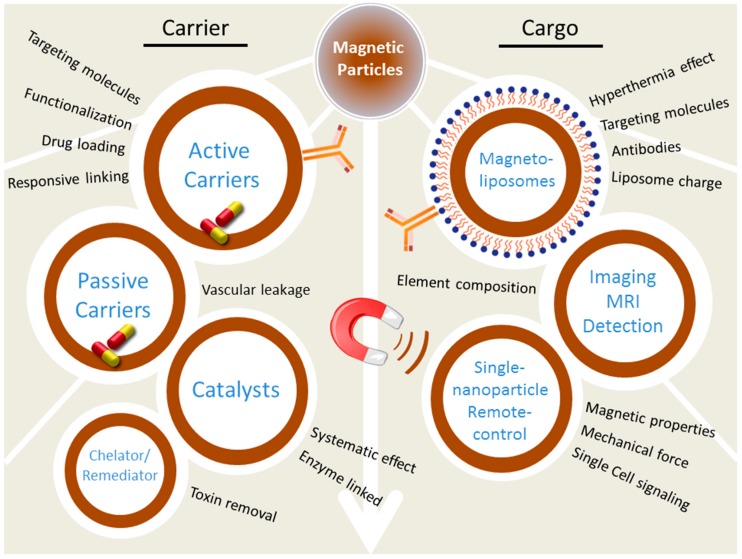
The scheme of magnetic particles utilization. MRI: magnetic resonance imaging.

During recent years, there has been much interest in novel materials in the area of nanotechnology. Magnetic nanoparticles belong to the group of nanotechnology-based materials with an impact in fields of analytical chemistry, biosensing, and nanomedicine. It has been nearly fifteen years since Pankhurst and colleagues wrote their famous review on magnetic nanoparticles in biomedicine [1]. More than 50 reviews with the phrase “Magnetic nanoparticle” in the title have been published in the last decade (according to the Web of Science). The four applications they have described for magnetic nanoparticles and microparticles aided in diagnosis and treatment of diseases in the following years. (i) Magnetic separation of biological entities contributed to the development of diagnostics. (ii) Magnetic nanocarriers contributed to drug delivery, while (iii) Radio frequency-controlled magnetic nanoparticles provided a new approach for cancer treatment, in addition to (iv) Magnetic resonance imaging (MRI) applications. The modification and functionalization of nanoparticles with various kinds of biomolecules, was already exploited at that time [2,3]. Nanomedicine introduced numerous novel materials for drug delivery and applications in molecular imaging where accurate detection is dependent on molecular signatures [4]. Magnetic particles are also applicable in the microscopic manipulation of nano- and micro-sized objects [5]. Applications of magnetic particles ranging from catalysis to drug delivery and remediation are only limited by their biocompatibility and immunogenicity, both of which can be controlled by proper layering and coating of particles (Figure 1) [6]. To shed some light on the rapid developments in this field and the future prospects, it is important to deal with the challenges associated with the processes of design, synthesis and characterization.

## 2. Magnetic Nanoparticles Design

### 2.1. From Physics and Chemistry to Nanomedicine

The design of magnetic nanoparticles for applications in nanomedicine is not easily implemented. There are many factors that should be taken into consideration at each step of the synthesis. These factors can dramatically change the expected outcome, yet they can be optimized in the early design steps. Both physical and chemical properties of particles can be controlled to fit various applications. Generally, magnetic nanoparticles are applied in nanomedicine and particularly in the field of cancer treatment [7]. Magnetic nanoparticle devices or magnetic composites are attractive for drug delivery due to their ability to respond to exogenous stimuli via a magnetic field [8]. This allows controlling drug release in spatial, temporal, and dosage controlled fashions. Nanocarriers can reach the tumor environment either passively through the leaky vasculature environment or actively using selective ligands [9]. Magnetic nanoparticles were used as a drug carrier for doxorubicin (an anthracycline antibiotic with antineoplastic activity) encapsulated into the apoferritin for targeted cancer therapy [10]. An MRI-based method was employed to control doxorubicin delivery via magnetic particles targeted using integrin ligands [11]. MRI applications also include MRI-guided cell replacement therapy and MRI-based imaging of cancer-specific gene delivery [12]. A liposome-based nanodevice used magnetic particles as carriers for DNA and drugs [13]. Paramagnetic nano- and microparticles were used for diagnostic detection of cancer biomarkers [14,15,16,17], viruses [18,19], and bacteria [20,21].

### 2.2. Physical Design

Magnetic properties are important for nanoparticles employed in externally controlled hyperthermia or heat induced by generated magnetic fields. The magnetic force can be utilized for movement and transportation of biological objects. The ability to drive a mechanical force within the cells is demonstrated to be useful for molecular level cell-signaling and controlling of cell fate. These capabilities have been used in applications of drug release, disease treatment and remote-control of single cell functions [22,23]. The size of magnetic nanoparticles is one of the major physical properties that can be utilized to tailor other properties such as magnetism and surface area. Controlled size synthesis of iron oxide nanoparticles has been explored by many researchers [24,25]. For example, organic-phase synthesis has been used to produce particles smaller than 20 nanometers. A later size increase was controlled by a seed mediated growth [24]. The major factors affecting particle size in the first step include the boiling point of solvent and reaction time [26]. Researchers coat particles to produce a stable system. The surface charge plays a role in maintaining repulsion between particles; however, it is important to optimize the ratio of inert to reactive compounds on the surface to ensure colloidal stability of the nanoparticles. Ho et al., described several elements that can be fabricated with iron oxide to achieve new physical and chemical characteristics [27]. For instance, gold-fabricated magnetic nanoparticles show desired magnetic as well as optical (from gold) characteristics. Metallic iron and cobalt-fabricated magnetic nanoparticles allow higher magnetization, yet the former requires coating to protect it from oxidation. Platinum-fabricated magnetic nanoparticles show great potential as contrast agents for both MRI and X-ray computed tomography (CT). On the other hand, porous magnetic nanoparticles have the same characteristics of solid nanoparticles yet they offer the additional opportunity to store and release drugs.

### 2.3. Chemical Design

There are two main strategies reported for magnetic particle functionalization. The first involves modification with bio-materials such as antibodies and oligonucleotides, and the second integrates the inorganic materials with other nanocomponents such as quantum dots [28]. In methods such as magnetic particle hyperthermia, the main challenge is to have a uniform distribution of nanoparticles and thus well-controlled temperature increase in tumor tissue. This can be achieved by targeting tumors with specific antibody-conjugated magnetic nanoparticles [29]. Magnetoliposomes, particularly those conjugated with antibodies, are able to deliver magnetic nanoparticles inside the tumor cells [30].

Ulbrich et al., suggested a modern view to the chemical design of magnetic particles for drug delivery purposes [31]. Briefly, they addressed the chemical modifications of particle’s surface in two categories: covalent and non-covalent modifications. Magnetic nanoparticles conjugated with drugs in a covalent fashion are useful to avoid cancer drug resistance and therapeutic side effects. Covalent bonds are required to be responsive in the cellular environment either by changes in pH [32], temperature [33] or simply to be cleavable by enzymes [34]. Non-covalent drug conjugation to nanoparticles has been exploited by researchers [31]. Successful drug delivery was reported using conjugate drugs to magnetic nanoparticles via hydrophobic interaction [35], electrostatic interactions [36], and coordination chemistry [37]. Biological molecules binding to magnetic nanoparticles such as nucleic acids are dependent on various interactions at the same time including electrostatic, hydrogen bonds as well as hydrophobic interactions [38]. During the process of design, it is very important to take these issues into consideration, particularly the strength of these binding interactions and the collective strength of various interactions at the same time. Much of the current research on magnetic nanoparticles is focused on studying such interactions for design and innovation of novel and desirable functions [31].

## 3. Magnetic Nanoparticles Synthesis

Previously, synthesis of magnetic nanoparticles was a crucial step in all studies dedicated to improving their properties and testing of their practical applicability. Nowadays, many of these nanomaterials are widely available commercially [39], thereby the need to synthesize these particles by individual teams or laboratories is eliminated, which ensures higher reproducibility of carried experiments. Despite this fact, the synthesis of nanoparticles remains the important step of studies focused on further examination, and is obviously indispensable for the development of new types of particles and new methods of their synthesis.

Problems of magnetic nanoparticles’ synthesis have in recent years attracted considerable attention, as is evident by several recently published reviews. The following text is therefore focused primarily on the reported synthesis of magnetic nanoparticles used for medical and biomedical applications.

**Figure 2:**
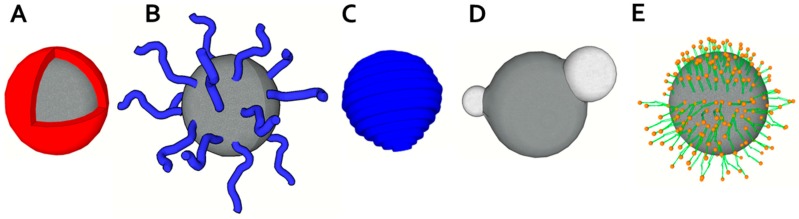
Structures of magnetic particles and their coating schemes. (**A**) Core-shell magnetic particle; (**B**) End-grafted polymer coated magnetic nanoparticle; (**C**) Magnetic particle fully encapsulated in polymer coating; (**D**) Heterodimer magnetic particle; (**E**) Hydrophobic magnetic particle encapsulated within lipid monolayer (upper part) and hydrophilic magnetic particle within lipid bilayer (modified from [41] with permission).

From the chemical point of view, materials commonly used for manufacturing magnetic portion can be divided into materials based on compounds (usually oxides) of iron [40], cobalt, nickel and some other elements usually combining several metals. These combined materials usually include materials based on copper, zinc, strontium, and barium. Magnetic nanoparticles also include the group of metallic nanoparticles and nanoalloys. Naturally, surface coatings of nanoparticles are an integral part of their synthesis [12,41]. Generally, the coating is designed to improve stability and solubility of nanoparticles, increase their biocompatibility, target-specificity, and to prevent from agglomeration, oxidation, corrosion, and toxicity [42]. Finally, the choice of core nature and surface modification is managed with regard to the intended use of the particles [1,12,42,43,44,45,46,47,48,49,50,51,52,53]. The use of bifunctional linkers (like avidin-biotin technique) is useful for attaching functional ligands with desired properties (“click” chemistry) and for further improving magnetic nanoparticle features [54,55]. Other examples of targeted surface modification of magnetic nanoparticles are those with the intended application potential for DNA detection or imaging techniques, typically X-ray Computed Tomography or MRI [51,56,57]. For surface modification of such nanoparticles several concepts using different metals such gadolinium or hybrid materials were used [47,58]. Moreover, magnetic nanoparticles with magnetic coating were described [53]. Several basic concepts of coating and functionalization of surface exist, including polymeric coating, liposomes and micelles utilization, and core-shell structures (Figure 2).

Several different methods can be used to synthesize magnetic nanoparticles with submicron diameter and with the same composition, e.g., according to the work of Grasset et al. [59] Y_3_Fe_5_O_12_ particles were prepared by the following different synthetic approaches: metal alkoxides hydrolysis [60], hydroxide coprecipitation [61], coprecipitation in microemulsion [62], glycothermal synthesis [63], glass-crystallization [64] or the citrate gel process [65,66,67], and some other related method [68]. There are many articles that extensively deal with the classification of methods of nanoparticles synthesis [28,43,44,69,70,71,72,73,74,75,76] even on an ultra-large-scale [77] or more usually in terms of synthesis and surface functionalization of particular types of magnetic nanoparticles, e.g., magnetic iron oxide nanoparticles [39,40,73,78,79,80] or MnO and NiO magnetic nanoparticles [81].

### 3.1. Iron-Based Magnetic Nanoparticles

**Figure 3:**
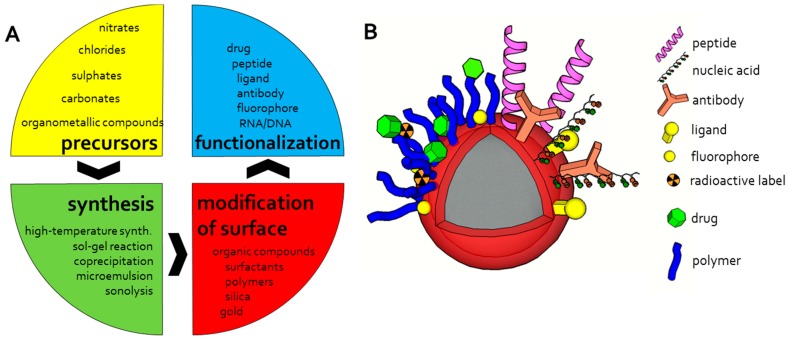
The scheme of magnetic particles design workflow (**A**) and possible modification and functionalization of magnetic particles (**B**).

Magnetic iron oxide nanoparticles represent materials with required magnetic properties mostly used for biomedical applications [78,80]. Significant advantages include primarily price, stability, and compatibility—magnetic iron oxide nanoparticles are inexpensive to produce, exhibit sufficient physical and chemical stability, as well as biocompatibility and are environmentally safe [41,53]. However, an important aspect is the possibility of their specific bioapplications that include magnetic separation, targeted drug delivery, MRI, magnetic fluid hyperthermia and thermoablation, and biosensing [82,83,84,85]. A broad listing of various methods of synthesis and surface modification of the magnetic nanoparticles based on iron has been exhaustively summarized by Wu et al. [40] and other authors (Figure 3) [39,41,78]. Briefly, these methods include both chemical synthesis approaches—mainly wet chemistry solution based methods [86,87,88,89,90,91], as well as physical one [92,93,94,95,96,97]. Exotic methods are presented more rarely, e.g., laser pyrolysis or chemical vapor deposition [76,78,98]. An integral part of the process of synthesis of magnetic nanoparticles is their surface modification that affects their properties and colloid suspensions stability [39,78]. Coating materials should have a high affinity to iron oxide core but must also meet the requirements for particles with respect to their function. Various approaches of surface modifications were brought by McCarthy and Weissleder in their review [99]. Step-by-step reproducible synthesis of new generation nanoparticles with a high control of magnetic core size, distribution, and hydrodynamic diameter was published by Mornet et al. [100]. These nanoparticles are based on maghematite core prepared by colloidal maghemite synthesis followed by covalent bonding of dextran macromolecules through aminopropylsilane groups and Schiff’s base. This process differs from the previously and commonly used one-step approach based on coprecipitation of iron (II) and iron (III) precursors in alkaline aqueous solutions of the hydrophilic macromolecule. Modification of this approach is the use of ultrasonic-assisted chemical coprecipitation which can provide the product with better dispersion and uniform size as described in the case of Fe_3_O_4_ nanosized cubic particles with a high level of crystallinity [101]. Another alternative way for magnetic Fe_3_O_4_ nanoparticles production may be the application of microwave synthesis [102]. Ecological and large-scale production of iron oxide magnetic nanoparticles allows an innovative approach that is based on using non-toxic and inexpensive reagents [77]. This method uses a mixed solvent (ethanol, water and hexane) for heating the mixture of iron chloride and sodium oleate to produce a waxy iron-oleate complex as a precursor for iron oxide nanocrystals preparation. Subsequently, the iron-oleate complex and oleic acid are dissolved at 1-octadecene and controllably heated. Poly(d, l-lactide-co-glycolide) coated superparamagnetic iron oxide nanoparticles prepared by the reaction of iron (III) acetylacetonate with 1,2-hexadecanediol, oleic acid, oleylamine and phenyl ether under reflux at 260 °C were successfully used for in vivo application as a contrast agent for MRI [103]. PEG-PEI/Fe_3_O_4_ nano magnetic fluid prepared by a coprecipitation method exhibit high plasmid pEGFP-C1 DNA loading efficiency due to the strong interaction with DNA phosphate framework [104].

A special category of magnetic nanoparticles based on iron represents particles having a combined composition or particles which contain a variety of metal dopants (usually M^2+^ cations, e.g., Zn, Mn, Co or Ni). These enhancements arose from the need to improve magnetic properties for applications such as molecular imaging. Metal-doped iron oxides with a spinel structure and formulas MnFe_2_O_4_, FeFe_2_O_4_, CoFe_2_O_4_, and NiFe_2_O_4_ were prepared by high-temperature reaction between corresponding divalent metal chloride and iron tris-2,4-pentadioate [105] but various other synthetic methods can be used [76]. While the particles with composition MnFe_2_O_4_ were found to be non-toxic in vitro, in the case of nanoparticles containing Co and Ni, their toxicity is a limiting factor for their use [41]. The size of the nanocrystal can be influenced by the choice of stabilizing surfactant(s), as it was demonstrated in the case of cobalt ferrite nanocrystal’s fabrication [106]. In this case, the complex (η^5^-C_5_H_5_)CoFe_2_(CO)_9_ served as a starting material; a mixture of lauric and oleic acid was used to produce monodisperse large cobalt ferrite nanocrystals. Smaller nanocrystals were prepared using hexadecylamine and oleic acid (different molar ratios of surfactant and precursor were used to moderate the size).

Mn–Zn ferrite magnetic nanoparticles used for heat-inducible gene expression were prepared through coprecipitation method and the surface of particles was covered by polyethylenimine (polyaziridine) [107]. Zinc-substituted ferrite nanoparticles Zn_0.9_Fe_0.1_Fe_2_O_4_ synthesized by a polyol method (zinc and iron acetates refluxed in the presence of tetraethyleneglycol) were used for heating glioma cells on hyperthermia assay [108].

Metallic iron nanoparticles include other separate and sometimes neglected group of magnetic nanoparticles. Their use for biological applications is partially limited due to their potential toxicity and their chemical instability. Hence, the metal used for the synthesis of these nanoparticles is easily oxidized; their surfaces are protected by coating with gold, silver or silica [56,57,109]. Synthesis of these types of nanoparticles is relatively difficult, but these particles have advantageous magnetic properties (e.g., high magnetization and superparamagnetism preservation). An example of this type of particles is Fe/Fe_3_O_4_ nanoparticles (FePt@Fe_3_O_4_), that is, iron nanoparticles covered with Fe_3_O_4_ shells [110]. Thermal degradation of iron pentacarbonyl Fe(CO)_5_ in octadecene at 180 °C and in the presence of oleylamine leads to a monodisperse of iron nanoparticles with a protective crystalline Fe_3_O_4_ shell [111]. Fe/Fe_3_O_4_ nanoparticles having an analogous composition with the possibility to moderate their size from 2 to 100 nm can be prepared using a nanocluster source and deposition chamber, where nanoclusters are coated with Fe_3_O_4_ shell [112].

Bimetallic or metal alloy magnetic nanoparticles represent another promising nanomaterial with superparamagnetic properties attractive for MRI. Iron-platinum (FePt) nanoparticles useful for biomedical applications have been prepared by different methods, such as solution phase synthesis or vacuum deposition [113]. Monodispersed and size tunable FePt nanoparticles can be prepared by the reduction of platinum acetylacetonate and decomposition of Fe(CO)_5_ while using oleic acid and oleyl amine as stabilizers [114]. Such nanoparticles were stable in a cell culture medium or phosphate buffered saline and exhibit the ability to bind DNA and proteins. Water solubility of FePt nanoparticles can be improved by further surface modification using carboxylate- and amine-based surfactants [115]. Furthermore, FePt nanoparticles can be encapsulated with shell based on cobalt sulfide (FePt@CoS_2_). Such nanoparticles exhibit cytotoxicity towards cancer cells [116,117]. On the other hand, coverage of FePt nanoparticles with a gold shell (FePt@Au) increases their biocompatibility [118]. Mentioned gold-coated FePt nanoparticles were synthesized from a high-temperature solution phase by the simultaneous reduction of platinum (II) acetylacetonate and decomposition of Fe(CO)_5_ in octyl ether solvent. Other types of binary metallic nanoalloys with advantageous magnetic properties are the nanoparticles containing iron and cobalt [119]. These nanoparticles are formed by a physical or chemical vapor deposition process (Fe_12_Co_88_, Fe_40_Co_60_, and Fe_60_Co_40_) that requires gold, silver or graphitic protective coating to prevent them from oxidation [120,121]. Two different ways were reported for gadolinium iron garnet particles Gd_3_Fe_5_O_12_ preparation (i) aerosol spray pyrolysis and (ii) drying of a mechanically grounded crispy product obtained by drying precursor solutions and further pyrolysis [122].

### 3.2. Cobalt Based Magnetic Nanoparticles

Examples of synthesis and bioapplications of magnetic nanoparticles of cobalt are much less common than iron due to cobalt toxicity. Except for the aforementioned metal-doped iron oxides with formula CoFe_2_O_4_ [105] and metal alloy magnetic nanoparticles (Fe_12_Co_88_, Fe_40_Co_60_, and Fe_60_Co_40_) [120,121], there are only a few works devoted to the preparation and/or theoretical bioutilization of magnetic nanoparticles of cobalt [1,123,124,125]. Commercially available carbon-coated cobalt nanoparticles were functionalized with polyhydroxy-, polyamine- or PEG_2000_-functionalized dendrons or polymers and designed for theoretical biomedical applications as drug carriers [123]. Unfortunately, no toxicity or tests involving the application of designed nanoparticles in vitro or in vivo have been described in that work. Stevenson et al., reported the synthesis of magnetic cobalt nanoparticle dispersions in biocompatible poly (dimethylsiloxane), dicobalt octacarbonyl Co_2_(CO)_8_ in the presence of block copolymer as starting materials. Such nanoparticles are promising materials for treating retinal detachments [124]. Particles exploited in another study were prepared in the same way, but similarly, they have not been tested for biocompatibility or toxicity [126]. Likewise, cobalt/silica carriers were investigated for their potential use in eye surgery to repair detached retinas [127,128]. A water-in-oil microemulsion reaction medium was used for the synthesis of hydrophobic molecule-based magnets composed of nanocrystalline cobalt complex salts (hexacyanoferrate, pentacyanonitrosylferrate) and chromium hexacyanochromate. Unfortunately magnetic properties have not been the subject of published studies [129]. A modified polyol-process was applied for crystalline cobalt nanoparticles with potential for applications in biomedicine [125]. Monodisperse cobalt nanocrystals were prepared by solution phase reduction of CoCl_2_ under high temperature in the presence of stabilizing agents [130].

### 3.3. Other Magnetic Nanoparticles

In typical applications ultrafine superparamagnetic particles based on Fe_3_O_4_ and γ-Fe_2_O_3_ are commonly used. Meanwhile other magnetic particles are studied and used exceptionally (except for the above-mentioned and rarely used particles containing cobalt). This is quite surprising according to the long known existence of other oxides and alloys with desired properties, e.g., Y_3_Fe_5_O_12_, SrFe_12_O_19_ or SmCo_5_ [131]. Some of these materials are hardly obtainable by conventional methods, but can be successfully prepared by new methods suitable for submicron particle preparation [59].

Preparation of two kinds of Ni based nanoparticles with different microstructure was reported and their magnetic properties were examined [132]. The first type of Ni nanoparticles consists of pure Ni nanocrystals with a carbon encapsulated Ni_3_C phase [133]. The second type represents nanoparticles with a Ni core and NiO coating on the surface [134]. Modified arc-discharge synthesis in a methane, argon, and hydrogen mixture atmosphere was performed. A water-cooled copper stage with a pure layer of the desired material served as an anode whereas a carbon rod was used as a cathode [48,132]. Generally, for the synthesis of NiO nanoparticles analogous methods can be used as mentioned above—spray-pyrolysis [135], a sol-gel process [136], polymer-matrix assisted synthesis [137], and thermal decomposition [138]. Some of the referred methods, however, do not guarantee desired features like sufficient homogeneity in size [139]. A low-cost coprecipitation method was used for the synthesis of pure and EDTA-capped NiO nanosized particles, while nickel chloride hexahydrate, sodium hydroxide, and ethylenediaminetetraacetic acid (optionally) served as reagents [139,140]. Preparation of another MnO and NiO nanoparticles exhibiting supermagnetism was performed using the decomposition of manganese or nickel cupferronate or acetate under solvothermal conditions [81].

## 4. Magnetic Nanoparticles in the Real World

Magnetic solids either of micro- or nano-size found many applications in the field of chemistry, biology and medicine. According to morphology, magnetic materials can be divided into two distinct groups. The first group represents micro/nanoparticles with a core-shell structure where the surface of pre-synthesized magnetic particles is modified. The second group includes composites of carbon materials or polymers with magnetic solids. In the next sections, real-life applications of magnetic particles will be discussed.

### 4.1. Analytical Chemistry—The On-Table Approaches

**Figure 4:**
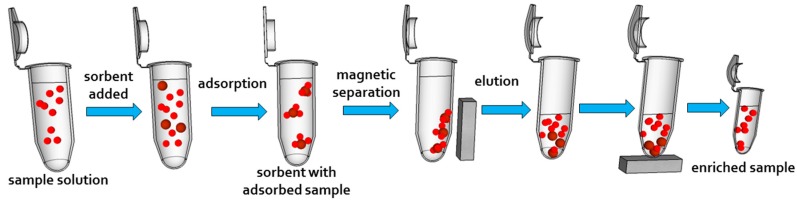
The scheme of an magnetic solid phase extraction procedure. Magnetic sorbent is added to the sample solution. Analyte is adsorbed on magnetic sorbent. The sorbent with adsorbed analyte is attracted using anexternal magnetic field. Subsequently, analyte is eluted from the magnetic sorbent and the enriched sample is analyzed.

Sample preparation techniques belong undoubtedly to key steps in almost all analytical procedures. Their aim is to isolate a target analyte from a complicated sample matrix, as well as remove analyte interferents and pre-concentrate analyte before analysis. Mentioned preparation techniques represent necessary steps, especially in the case of environmental and biological samples where target analytes are presented in trace concentrations and analytical instruments can hardly detect them directly. Magnetic solids are nowadays used as a common material for separation since they are cost effective and a highly efficient alternative to liquid-liquid extraction, coprecipitation/precipitation, ultrafiltration etc. solid phase extraction (SPE) attracted attention due to a high enrichment factor and its simple and fast operation. Nowadays, research on SPE is focused on the improvement of adsorbent materials. Safarikova and Safarik used the term magnetic solid phase extraction (MSPE), which exploits magnetic particles as an adsorbent for extractions [141]. In the case of MSPE, packing the sorbent within a column is not needed since separation is performed using an external magnetic field (Figure 4).

The performance of separation/preconcentration using magnetic particles depends mainly on the properties of magnetic core and functional cover. The size and shape of the magnetic core influence magnetic separation and convenient manipulation with particles. However, it is worth noting that the material should not retain any residual magnetism after magnetic field removal. The surface is responsible for the performance of the separation process and defines particle behavior towards surrounding environment including their affinity towards target ligand and their physicochemical stability within a solution. The broad nature of surface modifications ranging from chemical moieties to biological macromolecules determines the extent of target matter and specificity towards an analyte.

### 4.2. Preconcentration of Ions

Towler et al., pioneered the work on preconcentration of inorganic pollutants [142]. They used MnO_2_ modified magnetite microsized particles to remove Ra, Pd and Po from seawater. Although the study focused more on the environmental aspect than on analytical one, they paved the way for subsequent analytical works. For example, Wondracek et al., described magnetic core-shell silica structure functionalized with 4-amino-3-hydrazino-5-mercapto-1,2,4-triazole (Purpald) as a tool for SPE of Cu(II) from water [143]. Their material possessed an adsorption capacity which remained above 95% after 10 sorption/desorption cycles and had an enrichment factor of 98-fold suggesting use in analytical preconcentration procedures.

Cyclodextrins (CD) belong to cyclic oligosaccharides with a hydrophobic cavity and hydrophilic exterior. Organic molecules of proper size and shape can enter their cavity. Multiple hydroxyl groups of CD are known to provide compatibility with inorganic oxide surfaces via hydrogen bonding and can form complexes with ions. Magnetic particles modified with β-cyclodextrin were suggested to be suitable for organic (1-naphthylamine) and inorganic (Cu(II)) pollution cleanup and are promising for analytical separation/preconcentration [144].

The high surface-to-volume ratio, porous structure, hydrophobic nature and chemical characteristics make of carbon nanomaterial, especially single- and multiwall carbon nanotubes (SWCNT and MWCNT), makes it an ideal candidate for sorption of various inorganic/organic molecules and ions. Magnetic complexes with CNTs were reported for preconcentration/separation of U(IV), Cd(II), As(III), As(V), Ni(II), Pb(II), Zn(II) etc. [145,146,147,148,149]. However, sorption capacity and affinity of the analyte to CNTs can be improved by the attachment of additional ligands [148,150].

Dendrimers possess a highly branched 3D structure that results in high functional groups density. Amine-terminated poly (aminoamide) dendrimers (PAMAM) exhibit increased affinity for heavy metals. Dendrimers are considered as an effective sorbent material, however, theirs recovery by centrifugation or filtration is not practically applicable. The conjugation of dendrimers to magnetic materials was recently suggested to make their separation easier. Heavy metals can be coordinated to their numerous amine moieties. Protonation of amine moieties at low pH results in the release of heavy metal. Mentioned dendrimers are promising reusable chelating agents for heavy metal preconcentration [151,152,153]. Anbia et al., reported adsorption capacities of magnetic mesoporous silica modified with melanine-based dendrimer amines of 127.24, 125.80, 115.60 and 114.08 mg·g^−1^ Pb(II), Cu(II), Cr(VI) and Cd(II) metal ions, respectively.

### 4.3. Organic Compounds

Organic compounds are a broad group of environmental pollutants from which just several groups were selected to demonstrate the suitability of magnetic solids for their separation/preconcentration. Preconcentration of dyes and pharmaceutical products are mentioned here.

Dyes often possess a complex aromatic structure, making them hardly biodegradable. They are broadly used in industry and their ability to cause negative health effects even at low concentrations has caused a global concern. Nanomaghemite particles coated with sodium dodecyl sulfate to adsorb anionic and cationic dyes (Acridine orange, Methylene blue, Brilliant blue) were reported [154,155,156]. Surfactants can form various types of micelles on the surface of mineral oxides. In the case of magnetic particles dispersed in non-polar solvent, surfactants cover particles with one hydrophobic layer (polar head groups of surfactant are attached to particles and carbonic chains are exposed to solvent). However, in polar solvent, surfactants create bilayer around particles where polar heads are exposed to solvent and are attached to particles, too. This makes the magnetic solid modified with surfactants suitable for adsorption of amphiphilic compounds. Adsorption is mediated by hydrophobic interactions and electrostatic interaction of a surfactant polar head group and target ionic group of opposite charge. Electrostatic attraction of Methyl orange and MWCNTs decorated Fe_3_O_4_ nanoparticles modified with polyaniline were reported by Zhao et al. [157].

Pharmaceutical products represent the next group of analytes that deserve attention. Generally, high performance liquid chromatography is used for medical product analysis in various matrices (blood, urine, saliva, blood plasma, etc.); however, liquid-liquid extraction (LLE) or SPE is used for preconcentration and purification of an analyte. Previously, discussed modifications—polymers and surfactants—are also suitable for pharmaceutical compounds preconcentration [158,159,160]. In recent years, a selective recognition scheme of a target analyte using molecularly imprinted polymers (MIPs) combined with magnetic separation has been rapidly developing. MIPs are polymers formed in the presence of a target analyte which serves as a template. The template is subsequently removed and the remaining cavities provided lock-and-key compatibility with the analyte in shape, size and functional groups. Although MIPs mimicked biological receptors, they generally did not reach their high selectivity and affinity for targets. On the other hand, in comparison with biological molecules, they withstand harsh conditions and can be affordably obtained. Due to their thick imprinting layer bulk MIPs suffer from several obstacles like template leakage, complicated template elution or low adsorption capacity. However, imprinting sites on the surface of nanoparticles eliminate these drawbacks and bring several other advantages (see Niu et al. [161]). Magnetic particles modified with MIPs were previously used in the analysis of antibiotics and other drugs, hormones, dyes and pesticides [162,163,164,165,166,167,168].

### 4.4. Cells and Biomolecules

DNA isolation is one of the most important procedures in molecular biology, diagnosis and therapy, organism identification, etc. Phenol/chloroform extraction of DNA and its precipitation in ethanol is widely used for proteins and lipids removal from crude cell extracts [169]. However, this extraction method is laborious especially when analysis of several samples is required. DNA of certain quantity and also quality is required, since various inhibitors co-extracted with DNA can interfere with subsequent and frequently used polymerase chain reaction (PCR) amplification. Among others, SPE methods nowadays allow quick and efficient DNA extraction/purification and are found in most commercially available DNA extraction kits. The SPE of nucleic acids comprises four main steps as follows: cell lysis, nucleic acids adsorption to sorbent, removal of unattached molecules by washing, and elution of nucleic acids. The use of magnetic particles for nucleic acids SPE requires no centrifugation (especially needed for point-of-care devices). Adsorption, washing, and elution steps are obviously performed using buffers differing in pH and/or salt concentration and particles are manipulated using a magnetic field according to demands. Silica belongs to most the commonly used materials for DNA binding. Since amorphous silica possesses negative charge (silanol groups are deprotonated) at neutral pH, adsorption of polyanionic DNA on silica surface in plain water solution is not electrostatically preferred. However, the presence of highly concentrated chaotropic salts (guanidium thiocyanate, etc.) drives the adsorption of DNA on the silica surface while other macromolecules remain in the solution. Although this method has been used for many years, the mechanism of adsorption is still not fully understood. Li et al., summarizes that the main driving force for DNA adsorption of silica surfaces in chaotropic salt solutions can be shielding of negative charges, which weaken intermolecular electrostatic repulsive forces between DNA and silica, dehydration of DNA and silica as a consequence of water molecules strong capture by chaotrope, and creation of intermolecular hydrogen bonds [170]. After washing, which removes the remaining chaotropes, the elution of DNA is performed using buffers with low ionic strength and high pH. Vandeventer et al., showed that buffers without chaotropic salts can be also used for DNA adsorption on silica surface and suggested that buffer ions play more important beyond simply maintaining the pH [171].

Another strategy to support DNA interaction with magnetic particles is to induce positively charged moieties and promote electrostatic interaction with a negatively charged phosphate nucleic acids backbone. It has been demonstrated that surfaces modified with amino groups provide a positive charge in acidic conditions due to amino groups’ protonation and additionally provide electrostatic interaction without additional agents like chaotropes. It was recognized that the density of amino groups positively influences ability to adsorb DNA. Such an increase can be obtained using particles with a rough surface or amine-functionalized mesoporous silica magnetic particles [172,173]. Commonly, the sol-gel method is used for magnetic particle silanization. In subsequent steps, APTES containing a –NH_2_ group is induced. Alternatively, polyethyleneimine (PEI) as a molecule with a high density of amine moieties can be used for particle modification [174]. Polycharged magnetic particles modified by a surface-coating either with silica, polyvinylpyrrolidone or tripolyphosphate are easy to make in the laboratory and have demonstrated interactions with DNA for magnetic isolation applications [38]. Sequence specific isolation of nucleic acids can be performed using magnetic particles decorated with oligonucleotide probes which recognize target nucleic acid according to its sequence [175,176]. Adams et al., compared abilities of commercially available magnetic particles for isolation of RNA—silica coated, particles decorated with a polythymine oligonucleotide probe (oligo (dT)) and particles functionalized with an oligonucleotide complementary to target sequence [177]. Total mRNA was selectively extracted from the real sample spiked with target mRNA using oligo (dT). Silica coated particles isolated total RNA and particles with specific probe extracted mRNA highly enriched with the target sequence but exhibited significantly slower binding kinetics.

The previously discussed methods are used for peptides/protein enrichment through nonspecific hydrophobic interactions with carbon materials, silica, *n*-alkyl or mesoporous silica [178,179,180,181]. However, methods for specific peptide/protein isolation are rather developed. It can be achieved using antibody or MIPs immobilization on magnetic material [182,183]. Conversely, a hydrophilic surface of such particles is desired to minimize nonspecific adsorption. The imprinting of biomolecules is problematic in comparison with small molecules since they can denature during the fabrication process. Recombinant proteins with a polyhistidine tag can be efficiently purified using their affinity to metal ions like Ni^2+^. Although immobilized metal affinity chromatography (IMAC) and metal oxide affinity chromatography (MOAC) are standardly used for their purification, several magnetic materials exploiting this powerful affinity were reported [184,185]. Similar mechanism is used also for separation of phosphorylated protein/peptides [186,187].

Boronic acid can covalently bind to cis-diols in polysaccharides and glycoproteins and form five- or six-membered cyclic esters. Magnetic particles functionalized with boronic acid with advanced 3D-structure exhibiting high selectivity towards glycoproteins were reported [188]. Methods that exploit the high affinity of lectins for specific glycan structures and hydrophilic interactions (hydrophilic interaction chromatography, HILIC) were previously transferred from chromatography to use in the field of magnetic solids [189,190].

**Figure 5:**
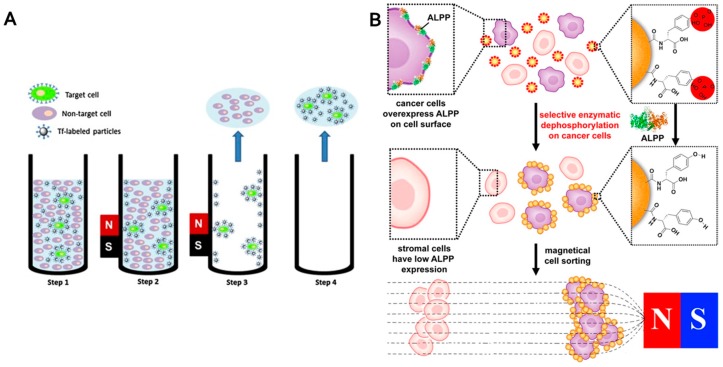
Different approaches to cells sorting. (**A**) Scheme of cell separation using magnetic particles. Target cells are bounded to magnetic particles modified with transferrin as a targeting moiety (step 1). Cells are magnetically separated (step 2). Non-targeted cells are removed with supernatant (step 3). Subsequently, target cells are resuspended and removed (step 4). (Reproduced with permission from [202]); (**B**) Enzymatic transformation of magnetic particles for selective sorting of cancer cells (reproduced with permission from [198]). ALPP: placental alkaline phosphatase.

Isolation of specific cell populations is relevant to study their specific genes or protein expressions. Currently, the most common method for target cells’ isolation is fluorescence activated cell sorter (FACS), however it is relatively expensive. Alternatively, magnetic solids can be used to separate eukaryotic and prokaryotic cells based on antibody-antigen affinity (immunomagnetic separation, IMS) (Figure 5A). Magnetic particles functionalized with antibodies can be used for separation of antigen-expressing cells [191]. IMS is suitable for enrichment of target bacterial cells and in combination with some progressive tools for bacteria identification. For example, matrix assisted laser desorption ionization-time of flight mass spectrometry (MALDI-TOF MS) can significantly shorten blood stream infection diagnosis by skipping blood culture methods [192]. Due to extremely high affinity and low-cost, bacteriophages were used as a biorecognition element for bacteria [193]. In comparison with antibodies, bacteriophages are more stable and the range of phages can be selected according to desired the target. Chen et al., reported immobilization of T7 bacteriophage on magnetic particles and used it for *E. coli* preconcentration [194]. Further, the affinity of antibiotics can be used to immobilize bacterial cells. For example, vancomycin is able to bind to the terminal peptide d-Ala-d-Ala of Gram-positive bacterial cell walls [195]. The preconcentration of *S. aureus* and *E. coli* using magnetic particles modified with β-lactam antibiotic amoxicillin was previously reported [196]. Carreira et al., reported magnetic separation of Gram-positive (*S. aureus*) and Gram-negative (*E. coli*) bacteria using cationized magnetoferritin [197].

The progressive sorting of cancer and normal cells based on the enzymatic transformation of a substrate immobilized on magnetic particles by an enzyme overexpressed on a cancer cell surface was reported by Du et al. [198]. These authors functionalized magnetic particles with d-tyrosine phosphate and ectophosphatases like placental alkaline phosphatase (ALPP) overexpressed on HeLa cells catalytically dephosphorylated phosphate-bearing magnetic particles. Subsequently, tyrosine modified particles selectively adhered to cancer cells and enabled their separation (Figure 5B). Although there are certain cancer cells which express normal levels of ALPP, further possibilities are offered by other genuine ectoenzymes identification [199].

Simple magnetic isolation of some cells is possible due to their inherent magnetic properties—red blood cells (hemoglobin iron is in a paramagnetic state), magnetotactic bacteria, etc. [200,201].

Magnetic materials provide several attractive utilities for sensing and biosensing. They are identical with advantages of magnetic separation/preconcentration but in this case, the analysis is directly performed on the magnetic solid or composite. In this field, magnetic solids can serve as a tagged solid support, be integrated into transducer materials or can be attracted from the sample by a magnetic field on the surface of the sensor [203]. Sensors/biosensors exploiting magnetic materials use different transduction principles, which will be discussed separately.

Electrochemical sensors/biosensors are one of the rapidly developing fields. Undoubtedly it is due to the progression in material chemistry and the demands for portable, un-expensive but sensitive analytical tools. In this case, biomolecules like antibodies, enzymes or oligonucleotides are immobilized on magnetic particles, which can be attached to sensing electrodes or serve as a transporter on electrodes. The topic of sensors and biosensors exploiting magnetic materials is so comprehensive that more focused literature is strongly recommended [204,205]. However, several electrochemical biosensors are described here.

Cancar et al., proposed a pesticide biosensor, which uses magnetic particles not for their magnetic properties but due to their easy synthesis and modification [206]. This enables covalent immobilization of enzymes and to maintain a suitable and extremely important orientation of an enzyme. They used a common transducer mechanism where organophosphate or carbamate pesticides, paraoxon and trichlorfon in this case, exhibit a cholinergic poisoning effect by the creation of complex (unstable in case of carbamates) in the active site of acetylcholinesterase [207]. If the enzyme activity is not inhibited, the substrate (acetylthiocholine) is converted to acetic acid and thiocholine. The latter is oxidized and detected on the electrode [208].

In addition, they covered the sensing electrode surface with a film of conjugated polymer poly(4,7-di(furan-2-yl)benzo[c][1,2,5]-thiadiazol). Polymers, especially conjugated polymers which possess an extended π-electron system, provide a suitable environment for the immobilization of biomolecules and often improve the sensitivity of the whole platform. On the contrary, Sarkar et al., electrodeposited magnetic particles decorated with poly(ethyleneglycol) on an electrode and attached a monoclonal antibody to *Vibrio cholerae* toxin using carbodiimide crosslinker chemistry and the remaining electrode surface passivated with bovine serum albumin [209]. Increasing the concentration of antigen was observed as increasing the charge transfer resistance (*R_ct_*) by an electrochemical impedance spectroscopy (EIS).

Leonardo and Campàs showed other possibilities that utilize magnetic materials for electrochemical analysis [210]. Firstly, they used a magnetic field to separate particles with an attached enzyme from a solution of enzyme surplus and subsequently used magnets placed under screen-printed electrodes to immobilize modified particles on a desired part of the electrode. Screen-printed electrodes are the most suitable for such applications since they possess a flat geometry.

**Figure 6:**
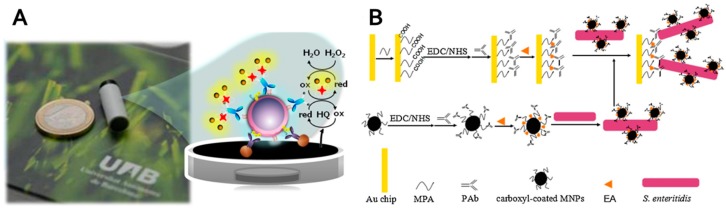
(**A**) Working electrode and scheme of target cell amperometric detection using horseradish peroxidase enzymatic activity, H_2_O_2_ as a substrate and hydroquionone (HQ) as an electron mediator; (**B**) The schematic representation of *S. enteritidis* detection using a magnetic particles-enhanced surface plasmon resonance sandwich assay. (Reproduced with permission from [211,212], respectively).

An interesting electrochemical magneto-actuated CD4^+^ T-lymphocytes biosensor was reported by Carinelli et al. [211]. CD4^+^ T-lymphocytes, which represent the primary target of HIV, were immunomagnetically separated using an antiCD3 receptor antibody. Although monocytes and macrophages express also CD3 receptors, separated CD4^+^ T-lymphocytes were identified using biotinylated antiCD4 antibody. Streptavidin-horseradish peroxidase (HRP) was incubated with cells attached on magnetic particles. The whole complex was immobilized on the surface of working electrode using a magnetic field. Amperometric detection using HRP enzyme, H_2_O_2_ substrate, and hydroquinone as an electron mediator between the enzyme and electrode was used (Figure 6A).

The work of Otieno et al., can demonstrate the possibility of magnetic solids use in the field of fluidic/microfluidic devices [213]. They reported a microfluidic device of high sensitivity for peptides connected with tumor progression (parathyroid hormone-related peptide, PTHrP) based on their direct capture on magnetic particles decorated with multiple peptide-specific antibodies and label enzymes from serum. After target immobilization, particles were washed and released into a detection chamber composed of 8 inkjet-printed electrode immunoarray. Wherein detection chamber, bedbound peptides are recognized by the second set of antibodies and simple amperometric detection mediated by HRP label was performed.

Magnetic materials are also broadly used in optical sensors and biosensors. They can be classified according to methods of readout (refractive index, chemiluminescence etc.). Chemiluminescence (CL), the emission of light during a chemical reaction, has a high potential for a variety of bioanalytical applications. Its few drawbacks, like poor selectivity due to interferents and low emission intensity, can be prevented by coupling with magnetic solids [214]. A chemiluminescence biosensor based analyte enrichment by magnetic capture of luminol was reported by He et al. [215]. They fabricated magnetic particles decorated with polydopamine and Au nanoparticles and attached an anti-leptin antibody to them as a capture antibody. Detection leptin antibody was assembled with hemin/G-quadruplex DNAzyme based on biotin-streptavidin interaction to create a sandwich-type immunocomplex with leptin. Biosensor high sensitivity was obtained due to signal amplification by DNAzyme luminol-H_2_O_2_ catalysis and magnetic enrichment. Surface plasmon resonance (SPR) is label-free detection mechanism when the refractive index of the sensor surface is monitored. SPR biosensor of *Salmonella enteritidis* based on magnetic particles modified with antibodies, which served for enrichment and amplification of SPR signal, was reported Liu et al. (Figure 6B) [212].

The readout can also be based on changes in magnetic particles size in the presence of target a analyte. It can be mediated by the simple attachment of a target on a single particle surface or by magnetic particles clustering in the presence of a target. Relaxation dynamics (Brownian relaxation time) of magnetic solids in a time-dependent magnetic field (oscillating, rotating, alternating, etc.) is connected with their hydrodynamic radius [216]. It was found that optic and magnetic anisotropies of magnetic particles and magnetic clusters are connected. For example, light absorption of magnetic particles and clusters of magnetic particles may differ when exposed to an oscillating magnetic field. Fock et al., compared the performance of a C-reactive protein (CRP) biosensor with a newly proposed optomagnetic and alternating current (AC) susceptibility readout [217]. Both methods detect agglutination of magnetic particles. However, magnetic susceptibility approach probes magnetic response of particles, and optomagnetic approach probes modulation of laser light transmitted through the sample. In comparison, optomagnetic readout enabled faster detection of particles aggregation and may reach higher sensitivity.

Although Fe_3_O_4_ magnetic particles were thought to be chemically inert it was shown that they possess enzyme mimetic activity similar to peroxidases [218]. This catalytic activity was subsequently used for the construction of colorimetric and electrochemical biosensors for glucose and H_2_O_2_ [219,220,221].

### 4.5. Therapy

In the field of therapy, magnetic particles possess several applications—hyperthermia, drug and DNA delivery exploiting magnetic field guiding and MRI. Required properties of such particles are broadly discussed in Issa et al. [222].

In modern medicine, the local increase of tumor tissue temperature (hyperthermia) is widely accepted as an effective adjunctive cancer therapy [223]. Several ways to increase tissue temperature to the required level using radio frequency, microwave or laser wavelengths was proposed. Hyperthermia is based on the fact that cells heated to a temperature >42 °C show sign of apoptosis and cells heated above 50 °C necrosis [224]. Despite this, the clinically relevant temperature that is needed to reach is still unclear and to reach temperature homogeneity in the target tissue is challenging due to physiological conditions like local perfusion variations [223]. The cancer cells are considered as more susceptible to heat than normal cells due to their higher rate of metabolism [225]. On a tissue level, tumors possess a disorganized vascular system and their ability to dissipate heat stress is decreased. Elevated temperatures also increase cell sensitivity to other treatments like radiation therapy or chemotherapy. In comparison with other methods magnetic particle hyperthermia enables local heating of the target tissue by embedding magnetic particles to the target tissue and by using an external alternating magnetic field to heat it. Magnetic particles are directly injected within the tumor body or in the artery supplying the tumor. Further, magnetic particles can be visualized using MRI so a combination of therapy and diagnosis is possible. Magnetic iron oxide (γ-Fe_2_O_3_, Fe_3_O_4_) particles are the most used materials for this reason due to higher biocompatibility than other materials (Ni, Co, etc.). Nevertheless, surface modification of such particles is necessary to prevent them from aggregation. Surface modification of magnetic particles influences their magnetization and specific absorption rate (measured in W·kg^−1^), the most important property for practical use (the higher specific absorption rate, the lower the injected dose to the patient). Commonly, the specific absorption rate of magnetic particles modified with SiO_2_ decreases by several tens of percent [226]. It was shown that the shape of magnetic particles also plays an important role during hyperthermia. In the case of ellipsoidal nanoparticles, heat release is increased due to the additional anisotropy shape and dynamic reorientation of rods [227]. It was demonstrated using a micro-tumor-like environment that in comparison with exogenous hyperthermia magnetic hyperthermia requires approximately 6 °C lower target temperature to produce same the cell death effect and exhibit more significant cytotoxic effects [228]. The heat is generated because of particles’ magnetic moment rotation due to an alternating magnetic field and friction of particles due to their oscillation.

As an alternative to magnetic hyperthermia, photo-thermal therapy based on nanoparticles with strong plasmonic properties was explored for the same purpose. The successful combination of plasmonic and magnetic properties provides materials with enhanced heating ability [229]. So called hybrid multifunctional (magnetic/photonic) particles (MMPPs) are based on magnetic particles decorated with Au or Ag nanoparticles (or vice versa) [229,230]. Au and Ag nanoparticles absorb light strongly in the Vis–NIR region and can induce heating by surface plasmas resonance. In addition, Au provides promising surface chemistry for modifications and in the case of Au presence in shells protects NPs against external agents. Synergic effects of heating can be also enhanced using loading of MMPPs with chemotherapeutic agents [231].

The systemic drug administration represents an issue in current medicine, especially in the case of drugs exhibiting cytotoxic effects like cytostatic drugs. The large doses needed to obtain a sufficient drug concentration within the pathological site and low drug specificity towards the target cause undesirable treatment side effects [232]. Site-specific drug delivery is a commonly accepted mechanism to preclude such complications. Magnetic targeting (drug immobilized on magnetic materials and targeting it using a magnetic field) is one possibility in this field. For such application, size, charge, and surface chemistry of particles are important since they influence blood circulation time and bioavailability. As Gupta and Gupta discussed, magnetic 10–100 nm particles are the most suitable for intravenous injection and perform the most prolonged blood stream circulation time [78]. Particles of a size higher than 200 nm are removed from the blood stream by mechanical filtration in the spleen and eventually by the phagocyte system. Smaller particles of size <10 nm are rapidly removed by extravasation and renal clearance. Not only surface of magnetic, but the surface of all colloidal particles influences their fate within living organisms. The stability of materials in physiological conditions is complicated by the presence of proteins and high ion concentration. For example, adsorption of counter ions can screen the repulsive surface charge of particles and decrease their stability. In addition, it is well known that proteins adsorb to solid materials. In the case of colloidal particles the title protein corona is being used [233]. However, the biological fluids are so complicated that the reason for particles instability can hardly be determined. Surface modification which makes magnetic particles stable in physiological conditions is highly researched area since particle aggregation can make treatment ineffective or can cause negative health effects. Myriads of magnetic particle preparation and functionalization procedures were reported, however only two types of particles entered clinical trials, i.e., particles coated with polysaccharides or silica [234,235]. Further, polymeric compounds are highly investigated to hide magnetic particles from the immune system. PEG is one of them since it is biocompatible, uncharged and hydrophilic, possesses low toxicity and immunogenicity, and can be prepared with a wide range of terminal functional groups and sizes. Other polymers like chitosan or polyethylenimine (PEI) were used for such reasons [236,237]. Sufficiently modified magnetic particles mostly exhibit no side effects on cell viability [238]. Targeting drug delivery can be based on passive, active or physical targeting. Magnetic particles can be used in all approaches. In the case of physical targeting, magnetic particles can be localized in a desired place by an external magnetic field; however, a low ability to recognize specific tissues is evident. Passive targeting is based on specific properties of a drug or its carrier and differences between a target site and healthy site. Typically, enhanced permeability and retention effect (EPR effect) can enhance localization of drug/carrier complex in tumors due to disrupted endothelial lining. Active targeting is based on the modification of targeting material with target-specific (bio)molecules, which increase the probability of its uptake by specific cells.

Targeted drug delivery based on magnetic particles was reported by Huang et al. [239]. The proposed magnetic particles were modified with two polymers—PEG and PEI. PEG was introduced due to its stealthy behavior and PEI provided the ability to conjugate targeting ligand for receptors overexpressed on cancer cells—folic acid in this case—and to interact with negatively charged cell membranes. Doxorubicin was loaded on modified particles by electrostatic attraction and hydrogen bonds. The acidic environment of tumors caused polymers protonation and drug release. Li et al., also reported pH-responsive behavior for γ-Fe_2_O_3_ particles modified with porous silica using rhodamine B as a reporter [240]. Paclitaxel, an anti-cancer drug poorly soluble in water, can be stabilized by a carrier based on l-arginine and human serum albumin and magnetically targeted [238].

Therapeutic delivery of nucleic acids into a patient’s cells as an alternative to drugs is of importance nowadays. However, low cell membrane translocation efficiency, short in vivo half-time and poor cell-specific targeting, complicates its broader application. In this field, delivery of microRNA (miRNA) is a highly investigated area. miRNAs are short noncoding RNA molecules which interact with target mRNAs to down-regulate or inhibit translation. Enhanced apoptosis of cancer cells induced by the combined effect of hyperthermia and miRNA delivery by magnetic particles was described by Yin et al. [241]. They targeted heat shock proteins Hsp70 and Hsp90 which protect cellular proteins from degradation and hinder of hyperthermia-induced apoptosis [242]. Mesoporous silica magnetic particles were used as a carrier for small interfering RNA (siRNA) for the down-regulation of vascular endothelial growth factor gene [243]. Further, they were capped by PEI-PEG cover to increase their biocompatibility and by fusogenic KALA Amphipathic Peptide (WEAKLAKALAKALAKHLAKALAKALKACEA) to increase their cell internalization.

**Figure 7:**
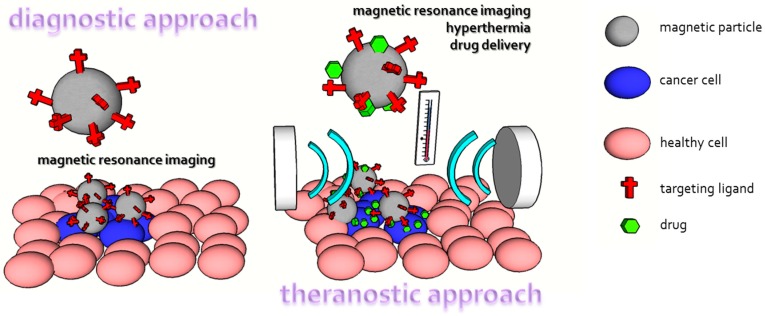
Comparison of magnetic particles utilization as a diagnostic and theranostic tool.

MRI is one of the most important diagnostic tools in medical science due to the high spatial resolution and soft tissue contrast. It mostly uses magnetic properties of hydrogen and its interaction with magnetic fields and radio waves (just like nuclear magnetic resonance) to produce images of living organisms. First, there is a high abundance of hydrogen in tissues (approximately 63%) and it possesses significant magnetic moments. Different molecular structures and hydrogen densities in tissues are responsible for specific proton behavior in magnetic fields and enable observation of contrast in images. It is worth noting that without contrast agents in most tissues, it is difficult to obtain information-rich images. There are two main groups of contrast agents—chelated lanthanide or transition metal ions (Gd^3+^, Mn^2+^) and paramagnetic particles. Iron oxide magnetic particles are the most frequently used magnetic-particles-based contrast agents. MRI contrast agents need to fulfill the following requirements: they need to improve contrast (induce large fluctuating magnetic fields), be biocompatible, and provide a suitable surface for targeting therapeutic molecules. The advantage of magnetic particles is that their biocompatibility has been highly explored (see Arami et al., for review [244]) and physiologic iron metabolism is responsible for their degradation. In addition, due to their (super) paramagnetic properties iron oxide magnetic nanoparticles strongly distort magnetic fields and create contrast exceeding their physical size. Undoubtedly, the current trend in medical research is to connect desired the properties of medicaments to obtain diagnostic and therapeutic (shortened as theranostic) tools. A convenient example is magnetic particles where drug delivery combined with hyperthermia or MRI is common (Figure 7) [240,245,246].

Magnetic materials are utilized in several fields. The magnetism phenomenon which enables their actuation provides easy, fast, and cost-effective separation of targets. The list of targets is broad and is not limited since several physico-chemical and biological approaches for target binding on magnetic materials are available. Such utilization in samples pretreatments in analytical chemistry is evident. Further, magnetic materials can serve as therapeutic agent carriers, MRI contrast agents for diagnosis, and for magnetic hyperthermia treatment of malignancies.

## 5. Conclusions and Future Perspectives

In conclusion, it must be mentioned that magnetic nanoparticles possess powerful potential in medicine, covering both, diagnostics and nano-based drug delivery. As versatile platforms, they can be simply functionalized for specific applications benefiting from their response to external magnetic fields. It is worth noting that one of the major biological procedures—isolation of DNA successfully utilizes magnetic materials. Additionally, many magnetic nanomaterials exhibit significant results in MRI, targeted-drug delivery or their combination—theranostics. Hence, we anticipate that magnetic nanoparticles belong to the “materials of the future”, which will significantly influence all fields of nanomedicine.
